# Italian pulmonologist units and COVID-19 outbreak: “mind the gap”!

**DOI:** 10.1186/s13054-020-03087-y

**Published:** 2020-06-29

**Authors:** Raffaele Scala, Teresa Renda, Antonio Corrado, Adriano Vaghi

**Affiliations:** 1 Pulmonology and Respiratory Intensive Care Unit, S Donato Hospital, Via Nenni, 20, 52100 Arezzo, Italy; 2 Respiratory and Critical Care Unit Cardio-thoracic and Vascular Department Careggi Teaching Hospital, Florence, Italy; 3 Pneumologic Center “Misericordia”, Sesto Fiorentino, Florence, Italy; 4 Italian Thoracic Society/Italian Association of Hospital Pulmonologists (ITS/AIPO), Florence, Italy

The outbreak of COVID-19 in Italy has shown the inadequacy of the health system to counterbalance a massive request for ICU care [1]. One fourth of > 1500 COVID-19 patients died after the admission in Lombardia ICUs; in only 11% of them, noninvasive ventilation (NIV) and/or high flow nasal cannula (HFNC) was attempted early to prevent respiratory deterioration and invasive mechanical ventilation (IMV). Conversely, in Chinese reports, NIV and HFNC were used respectively in between one third and two thirds of less severely hypoxemic COVID-19 patients keeping lower hospital mortality [2]. The success of noninvasive respiratory assistance in avoiding intubation is higher if attempted earlier in hypoxemic patients (PaO_2_/FiO_2_ > 150) [2]. Even after failure, NIV and/or HFNC may be good players to facilitate weaning from IMV and discharge from ICU. Clinical experts-guided hierarchical COVID-19 management strategy including intensivists and pulmonologists might have improved outcomes in some Chinese provinces [3].

The delayed admission in Lombardia overcrowded ICU of severely hypoxemic COVID-19 patients meeting the criteria for IMV without being offered a HFNC/NIV trial must have played a crucial role. Where should have been earlier and properly noninvasively supported acute patients with and without COVID-19 to keep the highest the ICU capacity?

Respiratory high-dependency care units (RHDCUs) are specialised cost-effective environments offering an “intermediate” level of care between ICU and ward, where NIV/HFNC, weaning from IMV and discharge of ventilator-dependent patients are provided [4]. Italian RHDCUs are mainly located inside the pulmonology ward and work following a step-up/step-down flexibility according to changes in clinical status. The “gap” between the Italian RHDCU network and pre-COVID-19 respiratory needs might largely explain ICU network failure in Lombardia [4]. A national survey performed at the beginning and 1 month after the COVID-19 outbreak demonstrated an increase rate (94% vs 12%) of Italian Pulmonologist Units (IPUs) accounting for 841 extra-beds involved in the fight against COVID-19. This was associated with the “up-grading” of 84% IPUs towards RHDCUs. Moreover, 72% of these extra-beds were dedicated to provide NIV/HFNC which avoided intubation/death in 40% of cases (http://www.aiponet.it/news/speciale-covid-19/2463-il-94-delle-pneumologie-e-in-prima-linea-nella-lotta-contro-l-infezione-da-covid-19.html) (Table 1). The expanded IPU network together with national more restrictive measures against virus dissemination after the Lombardia outbreak has contributed to the mitigation of COVID-19 impact on mortality in other regions.

**Table 1:** Distribution of RHDCU beds at the pre-COVID-19 time and of pulmonologist extra beds during the COVID-19 outbreak according to the different Italian regions

Regions	Population, inhabitants	Pre-COVID-19, E-RHDCU beds (min-max)	Pre-COVID-19, A-RHDCU beds	COVID-19, hospitalised pts*	COVID-19, ICU pts*	COVID-19, IPU extra-beds**	COVID-19, IPU NIV pts**
Lombardia	10,060,574	101–201	77	11,815	1330	378	240
Lazio	5,879,082	59–118	13	1079	154	0	0
Campania	5,801,692	58–116	18	468	126	26	4
Sicilia	4,999,891	50–100	16	484	75	39	12
Veneto	4,905,854	49–98	36	1633	356	63	10
Emilia-Romagna	4,459,477	45–89	61	3779	351	40	45
Piemonte	4,356,406	44–87	12	2985	452	63	29
Puglia	4,029,053	40–81	22	590	106	0	21
Toscana	3,729,641	37–75	49	1116	279	92	28
Calabria	1,947,131	19–39	8	130	18	24	8
Sardegna	1,639,591	16–33	0	113	24	0	0
Liguria	1,550,640	16–31	4	1142	175	37	0
Marche	1,525,271	15–31	4	998	167	28	12
Abruzzo	1,311,580	13–26	4	322	69	6	0
Friuli Venezia Giulia	1,215,220	12–24	14	229	60	13	17
Trentino-Alto Adige	1,072,276	11–21	7	584	140	31	5
Umbria	882,015	9–18	24	173	47	1	4
Basilicata	562,869	6–11	10	36	18	0	0
Molise	305,617	3–6	0	27	8	0	0
Valle d’Aosta	125,666	1–3	0	92	26	0	0
**Italy**	**60359546**	**604**–**1207**	**379**	**27795**	**3981**	**841**	**435**

PSN_2006_08_28_marzo.pdf

*NIV* noninvasive ventilation

A = RHDCU: active beds of respiratory high-dependency care units according to the 3rd Census of Italian RHDCU promoted by ITS/AIPO, updated to 15 February 2020 (rate of adhesion to the survey of IPU: 90.7%)

E = RHDCU: estimated needed beds of respiratory high-dependency care units according to the National Health Plan (2006–2008), http://www.salute.gov.it/resources/static/primopiano/316/

*Data from the Ministry of Health update to 30 March 2020, http://www.salute.gov.it/portale/news/p3_2_1_1_1.jsp?lingua=italiano&menu=notizie&p=dalministero&id=4362

**IPU: Italian pulmonologist unit; data of the first survey promoted by ITS/AIPO on the role of IPU in the midst of pandemics of the Pandemic (24 March 2020), ref. (http://www.aiponet.it/news/speciale-covid-19/2463-il-94-delle-pneumologie-e-in-prima-linea-nella-lotta-contro-l-infezione-da-covid-19.html)

In conclusion, what could we learn from the Italian COVID-19 outbreak? The Italian health system needs a stronger pulmonologists/RHDCUs “backbone” for the governance of “ordinary” burden of respiratory diseases to mind the gap against next unforeseen pandemia.

## Data Availability

Yes
